# Association of a *de novo *16q copy number variant with a phenotype that overlaps with Lenz microphthalmia and Townes-Brocks syndromes

**DOI:** 10.1186/1471-2350-10-137

**Published:** 2009-12-16

**Authors:** Tanya M Bardakjian, Adele S Schneider, David Ng, Jennifer J Johnston, Leslie G Biesecker

**Affiliations:** 1 Clinical Genetics, Department of Pediatrics, Albert Einstein Medical Center, Philadelphia, PA, USA; 2 National Human Genome Research Institute, National Institutes of Health, Bethesda, MD, USA

## Abstract

**Background:**

Anophthalmia and microphthalmia are etiologically and clinically heterogeneous. Lenz microphthalmia is a syndromic form that is typically inherited in an X-linked pattern, though the causative gene mutation is unknown. Townes-Brocks syndrome manifests thumb anomalies, imperforate anus, and ear anomalies. We present a 13-year-old boy with a syndromic microphthalmia phenotype and a clinical diagnosis of Lenz microphthalmia syndrome.

**Case Presentation:**

The patient was subjected to clinical and molecular evaluation, including array CGH analysis. The clinical features included left clinical anophthalmia, right microphthalmia, anteriorly placed anus with fistula, chordee, ventriculoseptal defect, patent ductus arteriosus, posteriorly rotated ears, hypotonia, growth retardation with delayed bone age, and mental retardation. The patient was found to have an approximately 5.6 Mb deletion of 16q11.2q12.1 by microarray based-comparative genomic hybridization, which includes the *SALL1 *gene, which causes Townes-Brocks syndrome.

**Conclusions:**

Deletions of 16q11.2q12.2 have been reported in several individuals, although those prior reports did not note microphthalmia or anophthalmia. This region includes *SALL1*, which causes Townes-Brocks syndrome. In retrospect, this child has a number of features that can be explained by the *SALL1 *deletion, although it is not clear if the microphthalmia is a rare feature of Townes-Brocks syndrome or caused by other mechanisms. These data suggest that rare copy number changes may be a cause of syndromic microphthalmia allowing a personalized genomic medicine approach to the care of patients with these aberrations.

## Background

Anophthalmia and microphthalmia (A/M) are part of a spectrum of ocular anomalies that includes coloboma. These conditions have a combined incidence of about 2/10,000 births [1-4]. The A/M spectrum may be isolated or syndromic and is etiologically and clinically heterogeneous with substantial phenotypic overlap in many cases. This makes accurate syndrome identification crucial in order to provide families and individuals with appropriate recurrence risk counseling.

One-third of individuals with A/M have associated malformations [4]. Both genetic and environmental causes of A/M have been noted. Mutations causing a phenotype that segregates in an autosomal recessive pattern have been found in *PAX6 *[5], *RAX *[6] and *CHX10 *[7] (MIM 607108, 601881 and 142993, respectively). Mutations in *SOX2 *(MIM 184429) [8,9] were recently described in individuals with A/M that segregates in an autosomal dominant pattern. Current estimates suggest that up to 15% of individuals with bilateral A/M have a mutation in *SOX2 *[9]. Bakrania et al., [10] also found individuals with deletions of *SOX2*. Mutations in *OTX2 *(MIM 600037) have been reported in up to 5% of individuals with A/M [9]. Mutations in *BCOR *(MIM 300485) have been seen in families with X-linked recessive inheritance of a phenotype strikingly similar to Lenz microphthalmia syndrome [11,12]. Lenz microphthalmia has cardinal manifestations of microphthalmia, growth retardation and limb anomalies [13]. Other malformations thought to be typical of this disorder include microcephaly, cognitive impairment, protuberant ears, missing upper central incisors, syndactyly, and a cylindrical thorax with sloping shoulders. However, it is clear that Lenz microphthalmia is clinically variable and genetically heterogeneous and prior data show that phenotypes compatible with this description map both to Xp and Xq, the former caused by mutations in the *BCOR *gene (MIM 309800) which causes a phenotype that overlaps extensively with Lenz microphthalmia, and shares the X-linked inheritance pattern in two families [12,14]. The Xq Lenz locus (MCOPS1 MIM 309800) has not yet been identified [15].

Here we report a patient with syndromic A/M that overlaps with Lenz microphthalmia syndrome (MIM 309800). Molecular evaluation of this patient showed a deletion of 16q, which includes the *SALL1 *gene, which causes Townes-Brocks syndrome (TBS, MIM 107480). This finding extends the notion of etiologic heterogeneity of syndromic microphthalmia and has implications for the approach to molecular diagnostics for this disorder.

## Case Presentation

The patient was born at full term weighing 6 lb 2 oz (~25^th ^centile) and length was 21 in (~75^th ^centile). He was apneic at birth and was intubated and transferred to the NICU. He was weaned from the ventilator at 7 days of age with no complications. Bilateral clinical anophthalmia was diagnosed on clinical examination. A cranial CT scan at one day of age showed mild prominence of the left lateral ventricle. A small cavum septum pellucidum was also noted. A focal area of parenchymal lucency was noted in the left parietal parenchyma. This raised the possibility of an *in utero *ischemic event. A CT scan of the orbits at 6 days of age showed some orbital contents in the right eye with an abnormal lens and soft tissue overlying the globe, and his clinical diagnosis was modified to left clinical anophthalmia and right microphthalmia. The left globe had a small oval focus of soft tissue, but this tissue could not confidently be determined to be optic. The optic nerves appeared severely hypoplastic. A cranial MRI done at 7 days of age identified absent optic nerves with a small optic chiasm. Renal ultrasound at 2 days of age showed the kidneys measured 3.7 cm bilaterally (normal 4.0-6.0 cm). Echocardiogram identified a small ventriculoseptal defect and patent ductus arteriosus, which both resolved spontaneously. He was also noted to have an anteriorly placed anus with a fistula that was corrected surgically at 2 months of age.

Clinical Genetics evaluation was performed at 5 years 9 months by one of the authors. Additional history at the time of this evaluation showed he failed to meet multiple developmental milestones. His height was 102 cm (<<5th centile; 50^th ^centile for 4 years), weight 29.5 pounds (<<5th centile; ~50^th ^centile for 2 years 6 months), and head circumference was 50 cm (10-25th centile). In addition to the findings described above, he was found to have apparently small, overfolded, and cupped ears, long trunk with narrow shoulders, low set thumbs, and partial cutaneous syndactyly of toes 4-5. Peripheral blood karyotype was performed at the referring institution and was reported as normal, 46,XY. The patient was diagnosed with Lenz microphthalmia syndrome (OMIM 309800 MCOPS1). Complete sequencing of coding exons and flanking introns of *BCOR *(the single known Lenz syndrome gene) was normal (data not shown).

He was recently re-evaluated at the age of 13 years. He was treated with melatonin to help with his sleep cycle. On examination, his weight was 65 lbs (<<5^th ^centile; ~50^th ^centile for 9 year old), Height 57 in (~10^th ^centile) and head circumference 52.5 cm (~10^th ^centile). In addition to the previously mentioned findings, he had multiple nevi on his chest, back, and limbs. All were 0.3 cm or less. He has made developmental progress but is in special education. Sequencing of *SOX2 *was normal (data not shown).

The array platform was of the oligonucleotide CGH type with average genome spacing of 37 kb and targeted probes in candidate gene regions with 5-20 kb probe spacing (for details see http://www.genedx.com/site/genomedx). Hybridization was performed on DNA isolated from peripheral blood leukocytes as previously described [16]. This assay identified an approximately 5.6 Mb deletion of chromosome 16. The deletion likely has a minimum boundary size of Chr16:45,018,886 - 50,571,154 (Human Genome Build 36) and includes 26 known genes (Table 1). This deletion was not present in the parents. The deletion of *SALL1 *was confirmed by a MLPA assay using the SALSA MLPA kit P180 (MRC Holland, data not shown). Assessment of 6 STRP markers on chromosome 16 showed inheritance of alleles consistent with biologic parentage. The proband is apparently homo/hemizygous at marker *D16S3396 *(Chr16:49,749,809 - 49,750,146) and does not share an allele with the mother suggesting the deletion occurred on the maternal chromosome. We conclude that this variant is *de novo *in the proband.

**Table 1:** Genes in the deleted region

Gene Name	Description	Transcription Start
*SHCBP1*	SHC SH2-domain binding protein 1	45,171,968
*VPS35*	vacuolar sorting protein 35	45,251,089
*ORC6L*	origin recognition complex subunit 6	45,281,058
*MYLK3*	myosin light chain kinase 3	45,293,694
*GPT2*	alanine aminotransferase 2	45,476,602
*DNAJA2*	DnaJ subfamily A member 2	45,546,774
*NETO2*	neuropilin- and tolloid-like protein 2	45,672,942
*ITFG1*	integrin alpha FG-GAP repeat containing 1	45,746,798
*PHKB*	phosphorylase kinase, beta isoform a	46,052,710
*ABCC12*	ATP-binding cassette protein C12	46,674,384
*ABCC11*	ATP-binding cassette protein C11	46,758,322
*LONP2*	peroxisomal LON protease-like	46,835,711
*SIAH1*	seven in absentia homolog 1 isoform a	46,951,946
*N4BP1*	Nedd4 binding protein 1	47,130,137
*CBLN1*	cerebellin 1 precursor	47,869,711
*ZNF423*	zinc finger protein 423	48,082,021
*TMEM188*	transmembrane protein 188	48,616,689
*HEATR3*	HEAT repeat containing 3	48,657,381
*PAPD5*	PAP associated domain containing 5	48,745,568
*ADCY7*	adenylate cyclase 7	48,879,323
*BRD7*	Bromodomain containing 7	48,910,441
*NKD1*	naked cuticle homolog 1	49,139,741
*SNX20*	sorting nexin-20	49,257,711
*NOD2*	nucleotide-binding oligomerization domain	49,288,550
*CYLD*	ubiquitin carboxyl-terminal hydrolase CYLD	49,333,461
*SALL1*	sal-like 1	49,727,386

## Conclusions

We report a boy with microdeletion of 16q11.2q12.1 identified by array CGH. Microdeletion of 16q11.2q12.2 has been reported as an emerging microdeletion syndrome [17]. That report includes two patients with microdeletions of 16q that overlap the deletion seen in the patient reported here. All three patients share similar centromeric endpoints (as defined by the last centromeric oligonucleotide detected as a single-copy) and two of the three patients share similar telomeric endpoints. The deletion in Patient 1 in Ballif et al [17] extended an additional 1.4 Mb towards the telomere when compared to the present patient. Other patients with deletions of 16q11q12 have been reported with limited molecular data (Table 2).

**Table 2:** Summary of patients with proximal 16 deletions.

Patient	Chromosomal Region	Methodology	Molecular deletion (minimal)	Hand/Foot malformation	Anal Malformations	Low Set/Dysmorphic Ears	Growth Retardation	Hypotonia	MR/DD
Present Case	16q11.2q12.1	oligonucleotide aCGH	Chr16: 45,018,886 -- 50,571,154	Low-set thumbs, partial 4,5 toe syndactyly	+	+	+	+	+
1A	16q11.1q12.1	550 band karyotype	ND	Proximally placed thumbs, short distal phalanges	-	+	-	-	+
1B (sister of 1A)	16q11.1q12.1	550 band karyotype	ND	Proximally placed thumbs, short distal phalanges	-	+	+	-	+
2	16q11.2q21	CGH/FISH	Chr16:49,728,524-62,004,665 (YAC 922f01-PAC LLNLP704M031126Q4)	Radial hypoplasia, preaxial polydactyly, malpositioned toes	+	+	-	NA	NA
3A	16q11.2q12.2	oligonucleotide aCGH	chr16:45,058,042-52,009,874	Malpositioned toes, syndactyly	-	+	-	+	+
3B	16q11.2q12.2	oligonucleotide aCGH	chr16:45,058,042-50,485,941	Malpositioned toes	Rectal prolapse	+	-	+	+
4	16q11.1q13	high resolution karyotype	ND	Malpositioned toes	-	+	+	+	+
5,6	16q12.1q12.2	650 band karyotype/somatic cell hybrids	chr16:49,028,856 (D16S308)	Malpositioned toes, long fingers	-	+ deafness	+	+	+
7	16q12.1q12.2	BAC aCGH/FISH	chr16:50,271,170-52,727,176 (BAC RP11-242n20-RP11-324d17)	Radial hypoplasia, syndactyly	-	NA	-	+	+
8	16q12.1q12.2	qPCR	Chr16:47,900,000-49,800,000 (approx.)	Long thumbs	+	+ deafness	-	NA	+
9A	16q12.1q13	850 band karyotype	ND	Malpositioned toes, syndactyly, small hands and feet	-	+ deafness	+	+	+
9B twin of 9A	16q12.1q13	850 band karyotype	ND	Malpositioned toes, syndactyly, small hands and feet	+	+	+	+	+

ND is not done, NA is not assessed, aCGH is array comparative genomic hybridization, BAC is bacterial artificial chromosome, FISH is fluorescence *in situ *hybridization, and qPCR is quantitative polymerase chain reaction.

References for Case numbers: 1 Hoo et al. [30], 2 Knoblauch et al. [31], 3 Ballif et al. [16], 4 Krauss et al. [32], 5 Schuffenhauer et al [33], 6 Callen et al. [34] (this patient was described in two papers), 7 Matthaei et al. [35], 8 Borozdin et al. [18], 9 Elder et al. [36]

The deleted region in the patient reported here includes 26 genes, including three recognized disease-causing genes (Table 1). This list includes the *SALL1 *gene, which when mutated causes Townes-Brocks syndrome [18]. Individuals with whole gene deletions have a milder phenotype than those who have dominant negative point mutations [19]. The classic phenotype for TBS includes imperforate anus, dysplastic ears with hearing impairment, and thumb malformations, although many affected patients do not have the typical phenotypic features. Less frequent manifestations of TBS include renal, heart, foot, and genitourinary anomalies, and mental retardation. This patient has a number of phenotypic manifestations that are not typical of TBS including A/M, cognitive impairment, borderline small head size, growth retardation, abnormal body habitus, and relatively normal thumbs. According to Kohlhase, the clinical diagnosis of TBS requires two of:

* Imperforate anus

* Dysplastic ears (overfolded superior helices, microtia)

* Typical thumb malformations (preaxial polydactyly, triphalangeal thumbs, hypoplastic thumbs) without shortening of the radius http://www.ncbi.nlm.nih.gov/sites/GeneTests/?db=GeneTests

The present patient only has one of these manifestations and therefore does not meet these stringent clinical criteria, though as noted above, the phenotype is acknowledged to be variable. One may speculate that an anterior-placed anus with a fistula is reminiscent of TBS, however it does not formally meet the criteria.

In contrast, the classical manifestations of Lenz microphthalmia are microphthalmia, growth retardation, and ear anomalies, all of which this patient manifested [13]. Clearly, this triad is not specific for Lenz microphthalmia and it is reported that > 90% have cognitive impairment [20], as seen in the present patient. A variety of skeletal and limb manifestations have been reported, although frequency data are not available.

Although some of the clinical features of the present patient overlap with the patients previously reported with 16q11.2 deletions and patients with TBS, the patient reported here had a major anomaly, A/M, which is clearly atypical for either of these molecular lesions. Microphthalmia has been reported in a pair of twins with TBS and a point mutation in *SALL1 *[21] and a patient from the pre-molecular era with a clinical phenotype consistent with TBS [22]. There are several possible hypotheses to explain this observation. First, it is possible that A/M is a very uncommon manifestation of TBS. Insofar as the twins reported by Botzenart et al are concerned, it is remarkable that although both twins had unilateral microphthalmia, neither their TBS-affected siblings nor their TBS-affected father had microphthalmia. It is reported that microphthalmia is two to five times more common in twins than in singletons [23,24], which limits the utility of this case in assigning causality of the microphthalmia in this pair of twins to the *SALL1 *mutation. Second it is possible that the deleted region in the patient reported here includes a gene, that when deleted, leads to A/M but with reduced penetrance. The prior report of a single patient with the co-occurrence of TBS and microphthalmia is intriguing [22] in this regard. As this patient has not be characterized molecularly and is now deceased (A.R. Cooper, personal communication), we cannot determine if that child had microphthalmia and TBS because of a 16q deletion or that s/he had a point mutation in *SALL1 *and microphthalmia is a rare manifestation of TBS. The third possibility is that in the present patient and the patient reported by Fraser and Cooper, [22] the 16q11.2q12.2 deletion is in *trans *with another variant, likely to be uncommon, that leads to a null for a putative A/M gene in this interval. It will be important to analyze additional cases with deletions of this region to distinguish these two hypotheses.

The deletion region also includes the genes *NOD2 *associated with Blau syndrome (OMIM 266600) [25] and *CYLD *associated with Brooke-Spiegler syndrome (MIM 605041) [26]. Missense mutations in *NOD2 *cause Blau syndrome and an increased susceptibility to Crohn's disease (MIM 266600) [27]. Blau syndrome is inherited in an autosomal dominant pattern and characterized by early-onset granulomatous arthritis, rashes, and camptodactyly. Ocular manifestations include uveitis, glaucoma, retinal detachment, and cataract [28,29]. No published reports of A/M and Blau syndrome were identified (negative results from PubMed using the two search strings: "anophth* AND Blau" and "microphth* AND Blau").

Heterozygous loss of function mutations in *CYLD *cause Brooke-Spiegler syndrome, which is characterized by a number of rare skin appendage tumors such as cylindroma, trichoepithelioma, and spiradenoma [30]. Early detection and aggressive treatment can prevent the significant deformity that can be caused by these tumors. These data suggest that the molecular delineation of this deletion in this patient present an opportunity to provide presymptomatic care that will significantly ameliorate the morbidity of this syndrome.

The patient reported here shares some clinical findings with the two previously reported patients [17] with deletion 16q11.2;12.2 namely; ear anomalies, toe abnormalities, hypotonia, and significant developmental delay. Interestingly, Patient 2 from Ballif et al., [17] was reported to have scattered pigmentary nevi, which was also found in the patient reported here.

We conclude from these data that the pleiotropic syndrome in this patient is caused by the deletion, which was detected by array CGH. The phenotype in this patient has some similarities to Townes-Brocks syndrome and to Lenz microphthalmia. The other suggestion from this report is that array CGH analysis should be considered in all patients with syndromic A/M. This is in addition to previous recommendations that such patients undergo chromosome analysis and *SOX2 *mutation analysis [9,10].

## List of abbreviations

A/M: anophthalmia or microphthalmia; CGH: comparative genomic hybridization; aCGH: array CGH; TBS: Townes Brocks syndrome;

## Consent

The parent of this minor patient has given written consent for the report to be published. The patient was also consented to a clinical research protocol reviewed and approved by the NHGRI IRB.

## Competing interests

The authors declare that they have no competing interests.

## Authors' contributions

TMB wrote the first draft of the manuscript. ASS and DN examined the patient and edited the manuscript. JJJ designed, interpreted, and performed some of the molecular evaluations, reviewed the literature, and wrote significant portions of the manuscript. LGB designed and interpreted some of the molecular evaluations, examined the patient, and wrote significant portions of the manuscript. All authors have reviewed and approved the submission of the manuscript.

## Pre-publication history

The pre-publication history for this paper can be accessed here:

http://www.biomedcentral.com/1471-2350/10/137/prepub
